# Superconducting artificial neural networks and quantum circuits

**DOI:** 10.3762/bjnano.17.51

**Published:** 2026-06-08

**Authors:** Anatolie S Sidorenko

**Affiliations:** 1 Technical University of Moldova, Chisinau 2004, Republic of Moldova

**Keywords:** artificial neural networks, high-performance computing, Josephson junctions, superconducting digital technologies, superconducting quantum circuits, ultra-low energy dissipation

Research on artificial neural networks (ANNs) is currently one of the most rapidly developing areas of modern science and technology [1]. Neural networks have demonstrated high efficiency in solving problems of pattern recognition and classification, clustering, forecasting, approximation, and optimization.

Artificial neural networks are commonly divided into two main classes: software-based and hardware-based implementations. Software neural networks are realised as programs running on conventional computing platforms and utilizing standard computational resources. Typical examples include photo- and video-based traffic violation detection systems or face recognition in crowds [2].

Hardware neural networks, in contrast, are implemented as specialised physical devices such as chips or processors [3]. The development of such systems represents a complex physical and engineering challenge; however, it enables significantly lower energy dissipation and higher computational speed compared to conventional von Neumann architectures. A representative example is the IBM TrueNorth processor implemented using standard complementary metal–oxide–semiconductor (CMOS) technology in 2014. This processor models fully connected and recurrent neural networks and integrates one million model neurons and 256 million synaptic connections [4].

For solving more complex computational tasks that require substantial processing power, a critical challenge arises: the need for a radical reduction in energy dissipation while simultaneously increasing performance. The future of high-performance computing with reduced energy consumption is closely associated with technologies characterised by ultra-low energy dissipation. A highly promising approach is the implementation of artificial neural networks using superconducting quantum circuits and superconducting digital technologies based on Josephson junctions. The energy consumption of a basic element of superconducting digital technology is on the order of 10^−19^ J, corresponding to up to seven orders of magnitude lower energy dissipation than that of semiconductor analogues [5]. Tuneable superconducting neurons and network architectures based on these principles have already been demonstrated [6].

Despite broad scientific and engineering interest in the development of artificial neural networks and quantum circuits, many fundamental problems remain unresolved. Among the most important challenges are: (i) processing of ultra-weak signals registered by highly sensitive cryogenic detectors, which is particularly relevant for deep-space communication and astronomical observations in the millimetre-wave and infrared frequency ranges [7–9]; (ii) cognitive radio and broadband signal reception, including superconducting signal processing for wideband spectrum sensing [10], as well as the development of quantum computing architectures and basic qubit technologies [11]; (iii) on-board signal processing in satellite systems, where severe constraints on power consumption and heat removal must be simultaneously addressed [12].

At present, the most energy-efficient circuit solutions are based on adiabatic superconducting logic. Such circuits do not exhibit a fundamental lower limit on energy consumption per operation. Adiabatic superconducting logic is actively developed worldwide, and recent demonstrations include a microprocessor with a total energy consumption of approximately 15 fJ per operation at a clock frequency of 5 GHz, including cooling overhead. This level of efficiency is comparable to the energy consumption of a single CMOS transistor [13].

The development of energy-efficient neuromorphic computers requires solving a broad range of fundamental, technological, engineering, and circuit-design challenges. In particular, both theoretical and experimental research is required to identify new physical phenomena suitable for novel base elements of superconducting neural networks, to develop advanced fabrication techniques for functional nanostructures, and to propose engineering solutions enabling the practical realisation of neuromorphic computing systems.

This thematic issue presents contributions from leading experts – theoreticians, experimentalists, technologists, and circuit designers – actively working in these research directions. Below, the main ideas and technical solutions discussed in this thematic issue are briefly summarised.

During the last decade, brain-inspired artificial neural networks based on superconducting elements have become the subject of intensive research [14–15]. A superconducting Gaussian neuron implemented as a two-junction interferometer and a detailed analysis of its transfer function are presented in [16]. A promising design of a neuromorphic ANN based on superconducting elements is proposed in [17]. The superconducting Josephson diode introduced in this work enforces unidirectional pulse propagation, prevents backward influence between connected neurons, and enables compact circuit designs with increased integration density.

The development of advanced technologies for the fabrication of layered functional nanostructures is a time-consuming process. To reduce the number of costly technological iterations, modelling of technological processes is essential for predicting the properties of prepared layers and multilayers. Smart computational experiments based on a mathematical model describing coordinated atomic motion predict unique magnetic properties of thin cobalt-based layers widely used in hybrid superconductor–ferromagnet structures for superconducting spintronics [18–19].

New elements of quantum circuits based on transmon “superconducting atoms” demonstrate rapid qubit control in real superconducting systems [20]. A theoretical study of the energy spectrum of a coupled single spin interacting with an infinitely coordinated Ising chain reveals a first-order phase transition between two stable phase-symmetric states, resulting in a splitting of the energy minima of the Ising chain. This phenomenon may be useful for engineering improved qubits for quantum computation [21].

Novel microwave quantum detectors [22], ultra-high-frequency receivers [23], and terahertz-range on-chip oscillators [24] based on superconducting heterostructures demonstrate significant potential for various applications of superconducting devices.

Geometry-controlled engineering of superconducting systems shows that geometric factors, such as interface curvature in normal metal–superconductor junctions, strongly influence the proximity effect and the decay length of Cooper-pair correlations [25]. For superconducting systems with complex topology, such as Fibonacci chains, enhanced spatial oscillations of the superconducting order parameter lead to a pronounced reduction of the critical temperature [26]. These findings are important for the design and optimisation of superconducting circuits, hybrid devices, and superconducting materials with complex and fractal topology [27–28].

Proximity-coupled superconductor/ferromagnet heterostructures demonstrate a number of novel phenomena, including the Fulde–Ferrell–Larkin–Ovchinnikov nonuniform superconducting state with singlet pairing [29], nonuniform superconducting states with triplet pairing [30], and fast vortex dynamics and relaxation processes in NbRe-based heterostructures, which may serve as a basis for devices requiring ultrafast relaxation times [31].

The concept of this thematic issue emerged during the 10th International Conference on Superconductivity and Magnetism (ICSM2025) and the 3rd International Conference on Quantum Materials and Technologies (ICQMT2025), held from 26 April to 3 May 2025 in Fethiye–Ölüdeniz, Turkey. The ideas, physical phenomena, technological approaches, and engineering solutions presented by invited speakers at these conferences constitute the scientific core of this issue.

As Guest Editor of this thematic issue, I thank all contributors and the Beilstein Journals Production Team for their professional editorial support. This work was supported by the HSE University project “International academic cooperation”. Financial support from the RM project (G.A. No. O2.02.01) “Nanostructures and advanced materials for applications in spintronics, thermoelectricity and optoelectronics” is gratefully acknowledged.

Anatolie S. Sidorenko

Chisinau, February 2026

## Data Availability

Data sharing is not applicable as no new data was generated or analyzed in this study.
